# Supplementary material for: Topic recognition and refined evolution path analysis of literature in the field of cybersecurity
Source: PLoS One. 2025 Feb 21;20(2):e0319201. doi: 10.1371/journal.pone.0319201 (PMC11844910; doi:10.1371/journal.pone.0319201)
Supplement: Attachment — Submitted filename: Response letter1222 --EN.docx [file pone.0319201.s001.docx]

**Description of the Revision**

Dear Editor:

We are very grateful for your constructive comments and suggestions for our manuscript (NO. PONE-D-24-13562). Due to the extensive nature of the revisions, we have highlighted the main modifications in red [to be reflected in the submitted paper]. The specific explanations for these changes are as follows:

Response to Reviewer 1 Comments

**Point 1:** The use the authors make of LDA is quite questionable. LDA provides a list of words for each topic with their probability but does not provide labels for the topics.

Where do the topic words in Table 1 come from? All the subsequent analysis is based on those words. If those labels have been assigned by the authors, a degree of subjectivity is present and may harm the validity of the results. The analysis of the evolution is fuzzy at its best.

**Response 1:** Thank you for pointing this out. We have carefully revised this comment. The revised section is highlighted in red. The modifications are as follows:

To identify the core research themes and their evolution across different stages in the field of cybersecurity, this study employed the LDA model for training and topic extraction from the literature. The LDA model analyzes a collection of documents to identify multiple topics, each represented by a set of words and their associated probability distribution. However, LDA does not automatically generate labels for these topics, requiring manual assignment based on the semantic meanings of the high-probability terms within each topic. For instance, in Topic 2 of Stage 2, the high-probability words and their probabilities are as follows: detection (0.073), cyberattack (0.050), cybersecurity_threats (0.033), forms (0.029), survive (0.023), cybersecurity (0.015), identification (0.015), spurious (0.012), avoid (0.011), and protecting (0.011). Based on the semantics of these high-probability words, the topic can be labeled as "Stage2_2: Detection of Cyberattacks." The topic words listed in Table 1 were generated following this procedure.

Additionally, the number of topics for each stage was determined by calculating the perplexity for each stage. To avoid overfitting, the value corresponding to the inflection point of the perplexity curve was chosen as the optimal number of topics. Using this method, the optimal number of topics for the ten stages was determined to be 9, 9, 8, 8, 9, 8, 7, 8, 7, and 7, respectively.

**Point 2:** Figure 4 is unreadable and appears as a mess of flows. The authors should provide a better explanation of this kind of representation.

**Response 2:** Thank you for pointing out the readability issue with Figure 4. We have redesigned the figure with the following improvements:

**Enhancement of Graphic Clarity:** We have redrawn Figure 4 to ensure its resolution meets the journal's requirements, thereby improving image clarity.

**Layout Optimization:** The arrangement of elements within the figure has been adjusted to present information more orderly, avoiding the previous clutter.

**Colors and Labels:** More intuitive colors and labeling schemes have been adopted to enable readers to distinguish different processes and data more easily.

**Subject Annotation:** Specific subject titles have been added within the figure to assist readers in understanding the content presented in Figure 4.

The revised Figure 4 is as follows:

**Point 3:** Also, the evolution analysis of topics should be examined through a quantitative framework, for example using the Jaccard index.

**Response 3:** Thank you for pointing this out. We have carefully revised this comment. The revised section is highlighted in red. The modifications are as follows:

In this study, we integrate topic modeling and word vector techniques to construct topic evolution paths by calculating inter-topic similarities and visualizing these paths using Sankey diagrams. Initially, we divide the time-series data into ten stages and apply the Latent Dirichlet Allocation (LDA) model to extract topics from the text data at each stage. By using the LDA model to the documents within each stage, we obtain the topic distributions for each period. After identifying the topics for each stage, we utilize word vector models such as Word2Vec to convert the keywords of each topic into word vectors. Subsequently, we generate vector representations of the topics through methods like weighted averaging.

To measure the similarity between topics across different time windows, we calculate the cosine similarity between topic vectors of adjacent stages. This approach determines the continuity and evolutionary relationships of the topics. Based on the computed topic similarity matrix, we set an appropriate similarity threshold (e.g., 0.50) to filter topic pairs with similarities exceeding the threshold, thereby establishing associations between topics in adjacent time windows. These associations form the topic evolution paths, reflecting the development and changes in topics over time, as illustrated in Figure 4.

**Point 4:** Also, it may be negatively impacted by the partial overlapping of some topic keywords (for example, what is the difference between "Detection of network traffic" and "Network traffic detection" in Table 2?).

**Response 4:** We are grateful for the reviewer's comments. During the thematic refinement process, we observed that although these two themes share the highest-frequency core terms, they exhibit significant differences in the composition of other high-frequency terms. Given this important finding, we carefully decided to distill two distinct themes to ensure their precision and representativeness. A similar situation was encountered in handling themes 5_2, 6_1, and 8_2. After a thorough review and incorporation of valuable feedback from experts, we recognized that our initial interpretation of certain aspects may have inadvertently led to a negative impact on the themes' evolutionary trajectory. In response, we conducted a rigorous reexamination and made necessary revisions, aiming to present the content in a more objective and balanced manner while avoiding any potential misunderstandings. This adjustment not only enhanced the accuracy of our analysis but also further strengthened the scientific rigor and credibility of the report.

Due to the extensive nature of the revisions, the specific changes are not detailed here. We have highlighted the main modifications in red in the submitted manuscript.

**Point 4:** The paper should be carefully revised for English, as there are many typos (e.g., txet instead of text in Figure 1) and wrong expressions (e.g., "this study divided of literature into ten stages" at the end of Section 3.1).

**Response 4:** We are grateful for the reviewer's comments. We have carefully reviewed the entire manuscript and revised it to correct all identified errors. Due to the extensive nature of the revisions, the specific changes are not detailed here. We have highlighted the main modifications in red within the submitted manuscript. Figure 1 has been revised. The incorrect expression "this study divided of literature into ten stages" has been revised to "this study divided the documents into ten stages" at the end of Section 3.1.

Response to Reviewer 2 Comments

**Point 5:** Why is the year 2023 not covered in this research review at least?

**Response 5:** Thank you for pointing this out. We have carefully revised this comment. The explanation for this problem is as follows:

This study covers the data collection period from 2003 to 2022. Due to the incomplete availability of data for the year 2023 at the time of analysis and manuscript preparation, we selected 2022 as the cutoff year for the data. This decision was made to ensure the integrity of the data and the accuracy of the analysis, thereby preventing potential biases or inaccuracies that could arise from incomplete data.

As discussed in the outlook section of the paper, we plan to continue the investigation into the evolution of themes in the field of cybersecurity in future research. Subsequent analyses will include data from 2023 and beyond. This ongoing data collection and analysis will allow us to track the latest developments in the research themes and verify the continued applicability of the conclusions drawn in the current study.

**Point 6:** The introduction is very simple and does not gradually transition from the general work environment to the specific research area that this manuscript concerns.

**Response 6:** We are grateful for the reviewer's comments. Due to the extensive nature of the revisions, the specific changes are not detailed here. We have highlighted the main modifications in red within the submitted manuscript.

**Point 7:** The research papers in the related works are not organized in order according to the year of publication (from oldest to newest). What are the contributions of the researcher through this manuscript compared to them?

**Response 7:** We are grateful for the reviewer's comments. Due to the extensive nature of the revisions, the specific changes are not detailed here. We have highlighted the main modifications in red within the submitted manuscript.

**Point 8:** In Figure 1 the texts need to be enlarged to be clearer.

**Response 8:** We are grateful for the reviewer's comments. Figure 1 has been revised as follows:

**Point 9:** The research papers used are very few and the researcher did not provide any comparison of the advantages and disadvantages of each method presented to clarify the vision.

**Response 9:** Thank you for pointing this out. We have carefully revised this comment. Due to the extensive nature of the revisions, the specific changes are not detailed here. We have highlighted the main modifications in red within the submitted manuscript.

**Point 10:** Use numerical values for results in conclusions and to explain proposed future work.

**Response 10:** We are grateful for the reviewer's comments. After careful consideration, we have added content about the number of topics in the form of a table and modified the conclusion. All revised sections are highlighted in red in the submitted manuscript. The relevant tables and conclusion sections are as follows:

This study employs the LDA2vc model and combines it with a phased approach to conduct an in-depth evolutionary analysis of cybersecurity research topics. It systematically reviews and analyzes the technological development path in cybersecurity, successfully outlining the context of topic development in this domain. The conclusions are as follows:

(1) In the analysis of topic evolution in the cybersecurity field, the LDA2vec model outperforms the DTM model. This study analyzes the thematic evolution in the field of cybersecurity and reveals that, although the DTM demonstrates relatively better topic coherence across most stages (for example, in Stage 1, the topic coherence score of the LDA model is -11.8 while that of the DTM is -12.2), and its score gradually increases over time, the limitations of the DTM in semantic recognition depth result in weaknesses in topic word identification (as shown in Figure 2). This indicates that the DTM has certain limitations in the accuracy and effectiveness of topic evolution paths. In contrast, the LDA2Vec model exhibits a more significant advantage in both topic differentiation and evolution path construction, effectively revealing the relationships and evolutionary trends between topics across different stages.

(2) The research topics in the field of cybersecurity exhibit characteristics of complexity and diversity. Through an in-depth analysis of the cybersecurity literature from 2003 to 2022, this study extracted 35 distinct topics that show significant trends of change over time. The proportion of literature related to each topic demonstrates noticeable fluctuations across different stages. For instance, the "Attack and Defense" topics maintained a high proportion of literature throughout all stages, increasing from 37.62% in Stage 1 to 50.52% in Stage 10. In contrast, the "Intrusion Detection" topics, while exhibiting an overall decreasing trend, demonstrate volatility: the proportion dropped from 23.35% in Stage 1 to 9.81% in Stage 8 but then rebounded to 12.39% in Stage 10. This fluctuation indicates that although "Intrusion Detection" was relatively less emphasized during certain stages, emerging security threats led to renewed attention in subsequent years. Particularly in later stages, the need for intrusion detection technologies to address novel attack methods resulted in a resurgence of research in this field. Furthermore, the proportions of literature related to "Traffic Analysis" and "Infrastructures" showed more stability but exhibited downward trends. Notably, "Identity" topics steadily increased from 22.06% in Stage 1 to 32.39% in Stage 10, reflecting ongoing interest and deeper research in this area. The "Data Protection and Privacy" topic, which became a focal point in 2007, saw its literature proportion rise from 1.80% to 7.91%, particularly driven by advancements in big data, cloud computing, and artificial intelligence, further intensifying research in this field.

(3) There is a dynamic and balanced relationship between technological development and security threats in the cybersecurity domain. With the continuous advancement of technology, certain research topics have experienced a noticeable reversal, indicating that early security issues have regained attention driven by new technologies. For instance, the topic "1_1 (Denial of Service) and 8_6 (DoS Attack Consequences)" accounted for 8.08% of the literature in Stage 1 but gradually declined to 4.02% by Stage 7. However, in the later stages, this topic rebounded to 4.76% in Stage 10, suggesting that with the application of new technologies and the emergence of novel attack methods, DoS attacks and their consequences have once again become a focal point of research. Similarly, the topic "1_4 (vulnerability) and 8_7 (Vulnerability)" had a literature proportion of 0.70% in Stage 1 and fluctuated in subsequent stages, reaching a peak of 1.27% in Stage 6 before experiencing a decline. However, starting from Stage 8, the proportion of this topic increased again, reaching 1.14% in Stage 10. This phenomenon demonstrates that, although some early security issues were neglected during technological progress, the emergence of new vulnerabilities has led to these issues regaining their focus in research. This reversal phenomenon reflects the sensitivity of the cybersecurity research field to the ever-changing threat landscape, as well as the interplay between technological innovation and security demands. Driven by new technologies, previously overlooked security issues have once again attracted attention, highlighting the intertwined relationship between technological advancement and security threats.

The research contributions of this paper are as follows:

(1) This study fully leverages the advantages of the LDA model in topic extraction and combines it with a phased division strategy. The analysis results effectively reflect the evolutionary patterns of research topics in the field of cybersecurity.

(2) This study identifies the key evolutionary stages in the field of cybersecurity, revealing the development trajectory from basic defense mechanisms to complex threat detection and response strategies. Specifically, the focus from 2003 to 2008 was on network infrastructure development and defense strategies, followed by a focus on intrusion detection and identity management from 2009 to 2016. From 2017 to 2022, attention shifted toward privacy protection and more advanced attack detection technologies.

(3) This study analyzes the development trajectory of topics in the field of cybersecurity from various aspects, including the evolution of attack and defense technologies, infrastructure maturity and expansion, refinement of identity and access management, data protection and privacy, intrusion detection techniques, and traffic analysis technologies. Additionally, while analyzing the evolutionary path, this research also examines phenomena such as merging, inheritance, division, generation, and disappearance based on Sankey diagrams. These findings reflect the changing research hotspots in the field of cybersecurity, providing not only a systematic perspective on literature evolution for cybersecurity research but also revealing shifts in research focus and trends.

This study has the following limitations:

(1) The depth of methodological integration needs to be strengthened. Although the LDA2vec model combines the advantages of the LDA and Word2vec models, the research lacks sufficient exploration of optimizing and adjusting model parameters.

(2) The evaluation criteria for the model should be more diverse. The current study conducts only a comparative analysis using the DTM model and primarily focuses on topic words and coherence indicators, leading to a singular set of evaluation criteria.

In future research, the research team will build upon the current study by using the latest datasets to explore the thematic evolution analysis in cybersecurity further. Specifically, future studies will optimize the LDA2Vec model by incorporating deep learning models, such as BERT and GPT, to enhance its ability to identify long texts and interdisciplinary topics. Additionally, we plan to introduce more cross-platform datasets (e.g., IEEE, Scopus) for comparative analysis to validate the generalizability and effectiveness of the methodology in different academic environments. The current study primarily conducts a comparative analysis using DTM; however, future work will consider incorporating more dimensional evaluation metrics, such as topic distinguishability and topic stability, to assess the performance of different topic models across stages more comprehensively. Through these improvements, we aim to provide richer data support and deeper insights, further aiding the understanding of the knowledge structure and development trends in the field of cybersecurity, thereby fostering continued progress and innovation in this area of research.

The proportion of literature in these areas at each stage is shown in Table 2.

**Table 2. Distribution of Literature Proportions by Research Perspective and Stage**

| **Research Perspective** | **stage1** | **stage2** | **stage3** | **stage4** | **stage5** | **stage6** | **stage7** | **stage8** | **stage9** | **stage10** |
| --- | --- | --- | --- | --- | --- | --- | --- | --- | --- | --- |
| attack defense | 37.62% | 39.61% | 38.01% | 37.26% | 39.23% | 44.85% | 44.30% | 46.07% | 47.60% | 50.52% |
| intrusion detection | 23.35% | 19.78% | 18.31% | 16.93% | 12.61% | 12.33% | 10.72% | 9.81% | 11.14% | 12.39% |
| traffic analysis | 1.00% | 1.81% | 1.79% | 1.37% | 1.76% | 2.01% | 1.44% | 1.35% | 1.21% | 1.16% |
| infrastructures | 0.70% | 0.62% | 0.54% | 0.66% | 0.52% | 0.66% | 0.56% | 0.65% | 0.67% | 0.69% |
| Identity | 22.06% | 26.45% | 23.18% | 23.28% | 26.66% | 25.55% | 25.07% | 25.86% | 28.74% | 32.39% |
| Data Protection and Privacy | 1.80% | 1.87% | 2.37% | 3.13% | 4.20% | 4.88% | 4.74% | 5.90% | 7.01% | 7.91% |

The topics with revisited phenomena are shown in Table 7, and their proportion of documents at each stage is shown in Table 8.

**Table 8. Proportion of Literature by Topic Revisited**

| **Topics** | **stage 1** | **stage 2** | **stage 3** | **stage 4** | **stage 5** | **stage 6** | **stage 7** | **stage 8** | **stage 9** | **stage 10** |
| --- | --- | --- | --- | --- | --- | --- | --- | --- | --- | --- |
| stage 1_1 stage 8_6 | 8.08% | 5.55% | 5.67% | 4.41% | 3.88% | 4.83% | 4.02% | 3.97% | 3.88% | 4.76% |
| stage 1_4 stage 8_7 | 0.70% | 0.81% | 0.71% | 0.97% | 0.92% | 1.27% | 1.17% | 1.04% | 1.08% | 1.14% |

**Point 11:** Ensure that all data for references is placed and their format is standardized.

**Response 11:** We are grateful for the reviewer's comments. Due to the extensive nature of the revisions, the specific changes are not detailed here. We have highlighted the main modifications in red within the submitted manuscript.

Thanks to the professional comments again that point out the above problems. The authors hope these explanations will answer your doubts.
